# Delayed uptake and intra-tree distribution of ^2^H-labeled irrigation water after repeated experimental summer drought in mature spruce compared with beech

**DOI:** 10.1093/treephys/tpaf153

**Published:** 2025-12-10

**Authors:** Benjamin D Hesse, Benjamin D Hafner, Timo Gebhardt, Stefan Seeger, Kyohsuke Hikino, Eva Stempfle, Regina Seiler, Karl-Heinz Häberle, Markus Weiler, Thorsten E E Grams

**Affiliations:** Department of Ecosystem Management, Climate and Biodiversity, Institute of Botany, University of Natural Resources and Life Sciences, Gregor-Mendel-Straße 33, 1180 Vienna, Austria; School of Life Sciences, Chair for Land Surface-Atmosphere Interactions, AG Ecophysiology of Plants, Technical University of Munich, Hans-Carl-von-Carlowitz Platz 2, 85354 Freising, Germany; School of Life Sciences, Soil Biophysics & Environmental Systems, Technical University of Munich, Hans-Carl-von-Carlowitz Platz 2, 85354 Freising, Germany; School of Life Sciences, Forest and Agroforest Systems, Technical University of Munich, Hans-Carl-von-Carlowitz Platz 2, 85354 Freising, Germany; Department of Crop Sciences, Soil Physics, Georg-August-Universität Göttingen, Grisebachstraße 6, 37077 Göttingen, Germany; Chair of Hydrology, Faculty of Environment and Natural Resources, University of Freiburg, Fahnenbergplatz, 79098 Freiburg, Germany; School of Life Sciences, Chair for Land Surface-Atmosphere Interactions, AG Ecophysiology of Plants, Technical University of Munich, Hans-Carl-von-Carlowitz Platz 2, 85354 Freising, Germany; Department of Forest Ecology and Management, Swedish University of Agricultural Sciences, Skogsmarksgränd 17, 907 36 Umeå, Sweden; School of Life Sciences, Chair for Land Surface-Atmosphere Interactions, AG Ecophysiology of Plants, Technical University of Munich, Hans-Carl-von-Carlowitz Platz 2, 85354 Freising, Germany; School of Life Sciences, Chair for Land Surface-Atmosphere Interactions, AG Ecophysiology of Plants, Technical University of Munich, Hans-Carl-von-Carlowitz Platz 2, 85354 Freising, Germany; School of Life Sciences, Chair of Restoration Ecology, Technical University of Munich, Emil-Ramann-Str. 6, 85354 Freising, Germany; Chair of Hydrology, Faculty of Environment and Natural Resources, University of Freiburg, Fahnenbergplatz, 79098 Freiburg, Germany; School of Life Sciences, Chair for Land Surface-Atmosphere Interactions, AG Ecophysiology of Plants, Technical University of Munich, Hans-Carl-von-Carlowitz Platz 2, 85354 Freising, Germany

**Keywords:** climate change, deuterium labeling (δ2H), drought stress recovery, forest ecosystems, soil water content, stable isotopes

## Abstract

Water uptake and distribution are critical for drought recovery, yet previous drought conditions have been shown to impair water transport by affecting soil–root contact and xylem conductivity. In order to investigate these dynamics, the approach of applying δ^2^H-labeled water as a controlled irrigation was adopted, with this irrigation being administered to a mixed stand of mature European beech (*Fagus sylvatica* (L.)) and Norway spruce (*Picea abies* Karst. (L)) trees in control (CO) and throughfall exclusion (TE) plots following 5 years of experimental summer drought. The δ^2^H concentrations were measured in soil, stem, twig and leaf water before and after rewetting to assess water pool turnover. The labeled water infiltrated the upper 70 cm of soil in both treatments within 48 h. However, a notable delay in water uptake and distribution was exhibited by TE trees in comparison with CO trees, where the label was detected in stems and leaves within 24 h. The TE beech demonstrated water uptake after 4 days, while TE spruce exhibited a more pronounced delay of 7 days. Despite this delay, TE trees exhibited a higher turnover of stem water pools (>75%) compared with CO trees (<50%), while leaf water turnover remained similar between treatments. The delayed uptake in TE trees may be attributed to fine root loss in both species and the suberization of surviving fine roots in spruce, which likely reduced water absorption efficiency. Additionally, the depleted stem water reserves in TE spruce may have delayed internal redistribution. These findings underscore the importance of considering species-specific recovery dynamics and provide valuable insights into the long-term impacts of drought on tree water relations.

## Introduction

It is beyond question that drought stress represents one of the most onerous challenges for forest ecosystems (McDowell et al. 2022). Due to the ongoing effects of climate change, with an increase in the frequency and duration of dry and hot periods (IPCC 2014), this challenge will become increasingly difficult to overcome for all ecosystems. In particular, ecosystems that have not been exposed to regular drought events in the past, i.e., in temperate regions, and have a long generation time will be threatened, as exemplified by temperate forests under the intense drought events of 2003, 2015 and 2018/19 in Central Europe (Leuzinger et al. 2005, Hartmann et al. 2018, Schuldt et al. 2020). It has been demonstrated that the dominant species of Central European forests, including Norway spruce (*Picea abies* Karst. (L)) and European beech (*Fagus sylvatica* (L)), are susceptible to drought (e.g., Tomasella et al. 2018, Leuschner 2020, Hesse et al. 2024, Paligi et al. 2025) and heat stress (e.g., Milad et al. 2011 and citations within). Nevertheless, numerous studies have been conducted on saplings/seedlings, which may not fully capture the effects of drought on a natural forest stand (Englund and Cooper 2003, Niinemets 2010). Mature trees may possess the capacity to mitigate the adverse effects of stress to a certain extent (Cavender-Bares and Bazzaz 2000, Betsch et al. 2011, Andivia et al. 2018, Groover et al. 2025). This can be achieved through, e.g., the utilization of stem water storage, which has been demonstrated to play a pivotal role in the resilience of temperate tree species (Köcher et al. 2013), the regulation of transpiration (Hesse et al. 2024) or changes in the root water uptake depth (Børja et al. 2017, Liu et al. 2023, Kinzinger et al. 2025). However, the internal water storage will probably be depleted following the occurrence of multiple severe drought episodes (Knüver et al. 2022), and its refilling will potentially further slow down the distribution of the newly absorbed water (Martinetti et al. 2025). Moreover, a shift in water uptake depth to deeper soil layers may occur (Brinkmann et al. 2019, Seeger and Weiler 2021, Bachofen et al. 2024), reducing the risk of drought-induced mortality but possibly delaying the uptake of precipitation during drought recovery. Spruce and beech exhibit a markedly disparate root distribution. Spruce has the majority of its roots in shallow soil, whereas beech has a generally more extensive root system at greater depths, as also observed on the experimental plots (Zwetsloot et al. 2019). However, both species show a certain flexibility in their rooting patterns under drought (Goisser et al. 2016, Nikolova et al. 2020, Zwetsloot and Bauerle 2021). In addition, the recovery phase, which occurs when water becomes available again after the drought period, has yet to be studied sufficiently. Drought damage often manifests only when plants cannot fully recover (Ruehr et al. 2019). Consequently, the recovery process is inherently energy- and resource-intensive with regard to water and carbon relations (Gallé et al. 2007, Ruehr et al. 2019, Hesse et al. 2023), e.g., for the repair of the water transport system (Brodribb et al. 2010, Tomasella et al. 2017) and regrowth processes (Trugman et al. 2018, Ruehr et al. 2019, Hikino et al. 2022*b*). In both aspects, beech seems to outperform spruce, with a quicker recovery, e.g., of stomatal conductance (Hesse et al. 2023), and less structural damage, i.e., loss of leaf area (Gebhardt et al. 2023), under recurrent drought. Once water becomes available again, it might be rapidly used in several processes, e.g., for transpiration or metabolism, and, therefore, have a fast turnover (stored water gets replaced and/or mixed with newly available water), especially in anisohydric species that do not rely much on safety mechanisms or storage refilling. To gain insight into the distribution of irrigation water added to the soil after a drought event, the subsequent uptake by trees, and the subsequent partitioning within the tree, we took advantage of the Kranzberg Forest Roof (Kroof) experiment (Grams et al. 2021). Following 5 years of repeated experimental summer droughts, the Kroof experiment started the recovery phase with controlled irrigation in 2019. The Kroof experiment focuses on two of the dominant tree species in Central European forests: the more isohydric Norway spruce, the most important species for foresters in Germany (Spiecker 2000, Hartmann et al. 2013), and the more anisohydric European beech, the dominant species in natural succession for Central Europe (Cavin et al. 2013, Pretzsch et al. 2013). We hypothesized that:

1. H1 Drought-stressed trees shift their mean water uptake depth due to the throughfall exclusion (TE) to deeper soil layers, with beech rooting deeper than spruce.
2. H2 Trees recovering from drought
  - take up the irrigation water on the day of irrigation with shallower-rooting spruce having access to irrigated water earlier than deeper-rooting beech;
  - distribute the irrigation water within the tree to the stem xylem and leaves quicker in beech than in spruce.
3. H3 Water pools in trees, i.e., stem and leaves, and soil under previous TE have a faster turnover time compared with unstressed trees and soils.

The distribution of water in formerly stressed (TE) and untreated (CO) plots was investigated using deuterated water (^2^H_2_O). Our attention was directed towards the soil, which had lost the majority of its plant-available water and exhibited a high degree of hydrophobicity (Grams et al. 2021). This suggests that the soil is expected to gradually refill with water from the top down (Brinkmann et al. 2018). As a subsequent step, the turnover of the various water pools was calculated using mixing models (Phillips et al. 2005) in order to ascertain the distribution of irrigation water and the duration of its residence in the different compartments of the soil–plant continuum (SPC).

## Materials and methods

### Experimental site and design

The Kroof experiment is located in the Kranzberg Forest near Munich in southeastern Germany (11°39′42″E, 48°25′12″N). The stand is composed of Norway spruce (*P. abies* Karst. (L.)) and European beech (*F. sylvatica* (L.)), planted in 1951 ± 2 AD and 1931 ± 4 AD, respectively (Pretzsch et al. 2014). The site benefits from a luvisol soil with a high water-holding capacity derived from loess of tertiary sediments and an average precipitation of 750–800 mm per year, which provides an abundant water supply (for details, see Grams et al. 2021). The clay content increases gradually with increasing soil depth, but the first 70 cm are characterized as silt with a medium clay content (Ut3). Groundwater is only available at a depth of ~7 m and is therefore not accessible to the plants. The Kroof Phase I experiment, conducted over a 5-year period (2014–2018), investigated the impact of recurrent summer drought on beech and spruce. The findings revealed significant disruptions in both species’ water and carbon relations (e.g., Tomasella et al. 2018, Hesse et al. 2019, 2021, Knüver et al. 2022). Also, belowground growth was influenced by the prolonged drought and less fine roots were produced in both species on the experimental site (Zwetsloot and Bauerle 2021). However, the fine root distribution hardly changed in both species even after 4 years of repeated summer drought (Zwetsloot and Bauerle 2021). A TE system was employed to induce drought stress, whereby all summer precipitation was withheld from six experimental plots. For comparison, six untreated control plots (CO, Grams et al. 2021) were established in close proximity to the TE plots. In 2019, the Kroof experiment entered a second phase (Phase II) involving the irrigation of the TE plots to the same level of soil water content as the CO plots (more details below). During the first 15 days after the irrigation, the roofs of the TE plots were kept closed. All data presented within this manuscript were therefore collected while the roofs were still closed.

### Relative extractable water in the soil and xylem sap flow density

Time-domain reflectometry (TDR) sensors (TDR100 and TDR200, Campbell Scientific, Logan, UT, USA) were installed to measure the mineral soil’s soil water content (SWC in vol%) beneath the litter layer. The TDR installations were made at three positions within each plot (Goisser et al. 2016, Grams et al. 2021). Four sensors were installed at four different depths to record the SWC down to 70 cm for each installation. The shallowest sensor recorded SWC at 0–7 cm depth, the second one at 10–30 cm, the third at 30–50 cm and the deepest at 50–70 cm, resulting in *n* = 6 for each depth, position and treatment (total of 144 sensors). The SWC was measured 9 and 1 days before (D−9 and D−1) and immediately after irrigation (D1, D2, D4 and D7). Thereafter, measurements were taken on a weekly basis. Based on the SWC, we calculated the relative extractable water (REW in %) using the permanent wilting point (Grams et al. 2021) and the maximum saturation values (Hesse et al. 2023) from the same experimental site for each soil depth.

Sap flow density per unit sapwood area was measured using thermal dissipation sensors (Granier 1987) at 10-min intervals. For each species and plot, sap flow was monitored in two trees at breast height, specifically in the outer xylem sapwood (0–2 cm depth). Two sensors were installed per tree, positioned on the north and south-facing sides of the trunk. The data from both sensors were averaged to obtain the mean daily sap flow density for each tree (SF in l dm^−2^ day^−1^). Measurements were conducted before, during, and after irrigation to assess changes in sap flow dynamics. Data shown here are from 7 days before the watering (pre, D−7 to −1), from the days on and directly after the watering (D0–7) and the week after (post, D8–15). A more comprehensive overview of the long-term recovery of xylem sap flow is available in Hesse et al. (2023) and during the drought period in Hesse et al. (2024).

### Labeling approach

Three CO and three TE plots were labeled with deuterated water during the irrigation process. To apply the labeled water, an irrigation system was designed with soaker hoses situated at a distance of 20–30 cm across the plot (CS Perlschlauch Premium, CS Bewässerungssyteme, Reichelsheim, Germany). Further details can be found in Grams et al. (2021). The CO plots were irrigated as well to apply the label and match the effects of saturated topsoil, temperature and nutrient availability (Grams et al. 2021). In order to ensure consistency in the irrigation treatment between the CO and TE plots, it was decided that 15 mm of water (~2034.5 ± 537.3 L) would be added to each CO plot with a deuterium signature (δ^2^H) of 1468.36 ± 44.47‰. The labeled water for the CO plots was mixed with 99.9% deuterated water (Carl Roth GmbH + Co. KG, Karlsruhe, Germany) in 1000 L tanks. The quantity of water appended to each TE plot was determined by utilizing SWC data. The mean volume of water added to the TE plots with a δ^2^H-signature of 289.30 ± 2.48‰ was 12,849.3 ± 2801.7 L, which is ~90 mm. The labeled water for the TE plots was mixed in a 60,000 L collapsible pillow tank (custom-made by FaltSilo GmbH, Bad Bramstedt, Germany) with 99.9% deuterated water (Carl Roth GmbH + Co. KG, Karlsruhe, Germany). The quantity of water applied to each plot was regulated by an electronic water meter (Wassermengenzähler, GARDENA Manufacturing GmbH, Ulm, Germany). The irrigation duration for the CO plots was ~7 h, while the TE plots were irrigated for ~40 h. Irrigation was conducted in three discrete campaigns, with one CO and one TE plot allocated to each campaign, over 3 weeks in May/June 2019. The irrigation of the plots commenced at 4 a.m. on Day 0. On the day of irrigation and the following days no rainfall occurred during daytime, except for D3 of campaign 3 (~8 mm during daytime). A few nighttime precipitation events occurred, with a max. precipitation of ~ 9 mm on D8 during campaign 1. Further weather information can be found in Table S1 available as Supplementary Data at *Tree Physiology* Online. For additional details on the calculations of the amounts of water applied and the irrigation procedure, see Grams et al. (2021).

### δ^2^H-signature in individual compartments of the soil–plant continuum

An overview of the samples collected for the assessment of δ^2^H excess in soils, stems, twigs and leaves is given in Table S2 available as Supplementary Data at *Tree Physiology* Online.

#### In soil water

Mineral soil samples (δ^2^H_soil_, Table S2 available as Supplementary Data at *Tree Physiology* Online) were collected using a Pürckhauer soil sampler (diameter: 2 cm and length: 100 cm) at several time points: before irrigation (D−6 and D−1), immediately after irrigation (D0, D1, D2, D4, D7), and for midterm observation on D15 and D22 (Table S2 available as Supplementary Data at *Tree Physiology* Online). For each plot, a single soil core was extracted from a depth of 70 cm, specifically beneath the beech, spruce and between the two species. Subsequently, each core was divided into seven 10 cm sections, with each section’s subsample collected in airtight 12 mL Exetainer vials (Labco, Lampeter, UK). Before sampling, the soil surface exposed to air was removed to minimize the evaporative enrichment of δ^2^H in soil water. Following each sampling event, the entire core was cleaned of residual soil, rinsed with tap water and dried.

#### In leaf and twig xylem water

Leaf samples (δ^2^H_leaf_) were obtained via the use of a canopy crane from the fully sun-exposed part of each tree crown, situated at an approximate height of 30 m. Approximately five beech leaves or 100 needles from a 1-year-old spruce shoot were collected in airtight Exetainer vials. Leaf samples were collected on the same days as soil samples (see above, Table S2 available as Supplementary Data at *Tree Physiology* Online). Twig samples (δ^2^H_twig_) were only collected on D−6 and D15 (Table S2 available as Supplementary Data at *Tree Physiology* Online). Twigs with a diameter of ~0.5 cm were stripped of bark, cut into pieces ~1 cm long and stored in Exetainer vials.

#### Cryogenic extraction of water from leaf and soil samples and isotopic analysis

All samples were stored at a temperature of 20 °C until cryogenic water extraction. Tissue water was extracted by cryogenic vacuum distillation for a period of 2 h (West et al. 2006). The extracted water was analyzed for its δ^2^H (against the Vienna Standard Mean Ocean Water (VSMOW) standard) with an isotope-ratio-mass spectrometer (IRMS) linked with a multiflow system (Isoprime, Elementar, Langenselbold, Germany; for details, see Hafner et al. 2017) against two monitoring standards (heavy: δ^2^H = 127.14‰ and light: δ^2^H—179.22‰, measurement precision—2.5‰ (1 SE of the standard measurements)). Before the extraction, right after extraction (including dried tissue) and after cleaning, the Exetainer vials with leaf samples were weighted for calculations of leaf water content (LWC— tissue water/fresh weight, in %).

#### In situ measurements of xylem water isotopes

In situ measurement of soil isotopic signatures and the stem water isotopic signature (δ^2^H_stem_) on one TE/CO plot pair was conducted using probes developed by Volkmann and Weiler (2014) in a measurement setup similar to the setups described in Volkmann and Weiler (2014), Volkmann et al. (2016) and Seeger and Weiler (2021). In contrast to the original setups, the probes were manually operated without valve manifolds. The respective active probe was manually connected to the flow controllers and a stable water isotope analyzer (L2130 i, Picarro, Santa Clara, CA, USA). The probes employ a measurement principle based on the equilibrium between the gas phase within a vapor-permeable membrane head (50 mm in length, 10 mm in diameter) and the surrounding liquid water. For a more detailed description, refer to Volkmann and Weiler (2014) and Volkmann et al. (2016). At DBH, in situ xylem water isotope probes were installed into stem-perpendicular, radially drilled holes (11 mm in diameter) into one (on the CO plot) or two (on the TE plot) beech and spruce trees. Each probe was measured once a day (from D0 to D15, Table S2 available as Supplementary Data at *Tree Physiology* Online) before midday against two monitoring standards (light standard: −74.22 ± 1.42‰ and heavy standard: 198.34 ± 2.87‰).

### Mixing models to calculate the turnover of water pools

We used two-endmember mixing models on both destructive and in situ sampled water isotopes to calculate the amount of labeled water in the respective tissues (Phillips et al. 2005, Hafner et al. 2020).

#### Xylem water and mean root water uptake depth

The mean water uptake depth of beech and spruce on the CO and TE plots was calculated from the δ^2^H_soil_ values before irrigation. In general, the δ^2^H value decreased toward the deeper soil layers in both treatments with only slight differences (see Discussion for a more detailed analysis). To achieve this, the δ^2^H_soil_ was plotted against the logarithmic soil depth and a linear regression was calculated for each plot individually. By applying the δ^2^H value of the xylem sapwood water (δ^2^H_stem_) to the regression formula, the mean root water uptake depth (RWU in centimeters) was calculated. The mean RWU on the CO plots was estimated to be ~30 cm in both species (Table 1), with beech exhibiting a slightly shallower depth (27.5 ± 11.4 cm) compared with spruce (33.5 ± 6.9 cm). On the TE plots, the RWU was observed to be shallower in both species (Table 1), with beech exhibiting a slightly shallower RWU (10.9 ± 7.6 cm) than spruce (15.9 ± 4.3 cm). In light of the RWU, we elected to utilize the soil δ^2^H values of 10–20 cm depth for the TE spruce trees and the mean δ^2^H value of 0–10 cm and 10–20 cm for TE beech trees as the δ^2^H signature of the mean water uptake depth (δ^2^H_water_uptake_). For CO trees of both species, a mean of the 20–30 cm and 40–50 cm ${\delta^2{\textrm{H}}}$ values was calculated and used as the δ^2^H_water_uptake_.


$$ \mathrm{\delta} {}{}^2{\mathrm{H}}_\textrm{water}\_\textrm{uptake}=\frac{\delta^2{\mathrm{H}}_{20-30\mathrm{cm}}+\left(\frac{\delta^2{\mathrm{H}}_{20-30\mathrm{cm}}-{\delta}^2{\mathrm{H}}_{40-50\mathrm{cm}}}{2}\right)}{2} $$

**Table 1:** Root water uptake depth (RWU, cm) of beech and spruce under control (CO) and throughfall exclusion (TE). *P*-value for treatment overall = 0.0085. Values are given as the mean ± 1 SD.

RWU (cm)	Beech		Spruce	
	CO	TE	CO	TE
	27.5 ± 11.4	10.9 ± 7.6	33.5 ± 6.9	15.9 ± 4.3

δ^2^H_water_uptake_ was used as one endmember (δ^2^H mix-signature of the soil water taken up by the tree) in the mixing model for the twig water and the other was the δ^2^H-signature of the twig/stem water before the start of the irrigation (δ^2^H_twig/stem_ of D−6) to calculate the fraction of labeled water in the twigs and stems (*F*_twig/stem_ in %).


$$ {F}_{\mathrm{twig/ stem}}=1-\frac{\mathrm{\delta} {}{}^2{H}_{\mathrm{twig/ stem}}-\mathrm{\delta} {}{}^2{H}_{\mathrm{water}\_\mathrm{uptake}}}{\mathrm{\delta} {}{}^2{H}_{\mathrm{twig/{stem}}_{D-6}}-\mathrm{\delta} {}{}^2{H}_{\mathrm{water} \_ \mathrm{uptake}}}\ast 100 $$

#### Leaf water

For the leaf data, we had to consider the evaporative enrichment of leaf water (Farquhar and Cernusak 2005, Werner et al. 2012, Cernusak et al. 2016). Therefore, we calculated the direct evaporative enrichment (EER in ‰, Table 2) and evaporative enrichment relative to the xylem water (ΔLW, Table 2) of the leaf water on D−6 and D15.


$$ \mathrm{EER}=\delta{}{}^2{H}_\textrm{Leaf}-\delta{}{}^2{H}_\textrm{Twig}\&\Delta \mathrm{LW}=\frac{\delta{}{}^2{H}_\textrm{Leaf}-\delta{}{}^2{H}_\textrm{Twig}}{1-\delta{}{}^2{H}_\textrm{Twig}} $$

**Table 2:** Daily xylem sap flow density (SF in l dm^−2^ day^−1^) of beech and spruce under control (CO) and throughfall exclusion (TE). Values are given as the mean ± 1 SD.

Species	Beech				Spruce			
Day	D−6		D15		D−6		D15	
Treatment	CO	TE	CO	TE	CO	TE	CO	TE
LWC [%]	52.7 ± 7.5	51.9 ± 7.1	50.1 ± 1.5	52.0 ± 0.9	51.8 ± 0.9	51.3 ± 13.1	51.1 ± 0.7	49.9 ± 1.4
EER [‰ ^2^H]	43.9 ± 7.9	37.7 ± 6.9	46.1 ± 9.9	40.7 ± 5.8	50.3 ± 7.1	45.1 ± 5.9	42.3 ± 4.9	39.4 ± 4.4
ΔLW	0.6 ± 0.1	0.6 ± 0.1	4.8 ± 12.8	−1.9 ± 3.3	0.7 ± 0.08	0.6 ± 0.07	2.1 ± 1.9	1.4 ± 0.6
δ^2^H_twig_ [‰]	−71.2 ± 8.1	−65.2 ± 11.3	−6.2 ± 24.2	20.6 ± 30.9	−75.5 ± 4.5	−72.1 ± 6.0	−6.4 ± 21.6	−31.6 ± 10.6

Evaporative enrichment was not different between D−6 and D15 for each combination (Table 2). We used the ERR corrected values of D−1 leaf water as one endmember and δ^2^H_water_uptake_ to calculate the fraction of labeled water in the leaves (*F*_leaf_ in %).


$$ {F}_{leaf}=1-\frac{\Big(\mathrm{\delta} {}{}^2{\mathrm{H}}_\textrm{leaf}- EER\Big)-\mathrm{\delta} {}{}^2{\mathrm{H}}_{\textrm{water}\_ \textrm{uptake}}}{\Big(\mathrm{\delta} {}{}^2{\mathrm{H}}_\text{leaf D-1}- ERR\Big)-\mathrm{\delta} {}{}^2{\mathrm{H}}_{\textrm{water}\_ \textrm{uptake}}}\ast 100 $$

#### Soil water

The values of D−1 and the irrigation water (δ^2^H_tank_, for CO: 1468.36 ‰ and TE: 289.30 ‰) were used as endmembers for the soil water samples. The fraction of labeled water (*F*_soil_ in %) was calculated for each depth, position and day.


$$ {F}_{soil}=1-\frac{\mathrm{\delta} {}{}^2{\mathrm{H}}_\textrm{soil}-\mathrm{\delta} {}{}^2{\mathrm{H}}_\textrm{tank}}{\mathrm{\delta} {}{}^2{\mathrm{H}}_\text{soil D-1}-\mathrm{\delta} {}{}^2{\mathrm{H}}_\textrm{tank}}\ast 100 $$

### Statistical analysis

For the statistical analyses, the isotopic data were expressed relative to the SLAP2 isotopic standard for ^2^*H* (IAEA 2017); isotopic data given in text and figures are calculated against the V-SMOW standard. Additionally, we calculated the δ^2^H changes by subtracting the mean of D−6 and D−1 from the values of individual time points (Δδ^2^H). Six trees on three plots were sampled for each treatment, day and species, giving six replicates for each measurement point in trees and three replicates for soil samples. Data were analyzed for statistical differences using R (R Development Core Team 2008) in RStudio (RStudio Team 2015). Data were plotted with the ‘ggplot’ function (package: ggplot2, version: 3.1.0) or the boxplot function (package: graphics, version: 3.5.2). Data were tested for homogeneity of variances (Levene test) beforehand, and the residuals of every model used were tested for normality (Shapiro test/Q-Q-Plot). For differences in the δ^2^H-signature of water extracted from soil, stem, twig and leaf, a linear mixed-effect model (‘lme’ function) was calculated, using the day, species and treatment as fixed and the tree individual nested in the plot as a random effect (package: nlme, version: 3.1–137). If the mixed-effect model showed significant effects, a post hoc test with the ‘emmeans’ function with Tukey correction (package: emmeans, version: 1.3.1) was performed. Data in text and tables are presented as means ± 1 SD.

## Results

### Changes in the volumetric relative extractable water upon irrigation and xylem sap flow density

**Figure 1:**
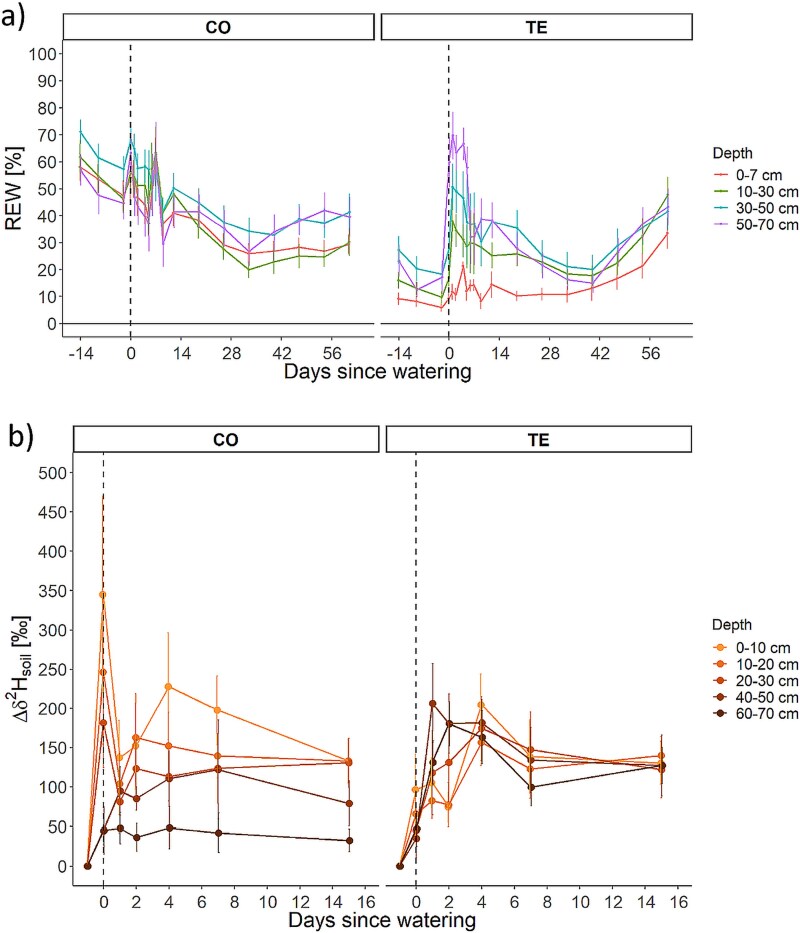
(a) Relative extractable water (REW in %) in control (CO) and throughfall exclusion (TE) of different soil depths (0–7 cm—orange, 10–30 cm—green, 30–50 cm—blue and 50–70 cm—purple, vertical dashed line—day of irrigation) and (b) soil water deuterium excess (Δδ^2^H_Soil_) based on values of D−1 for CO and TE of different depths (light orange—0–10 cm, …, dark orange—60–70 cm). Symbols show the mean ± 1 SE.

Prior to irrigation, the REW in the CO plots was ~ 49% in the deepest layer (50–70 cm) and 53% in the shallowest layer (0–7 cm) (Figure 1a). The TE plots exhibited an REW less than half of that observed in the corresponding soil layers of the CO plot (Figure 1a). The top layer on the TE plots exhibited exceptionally low REW, with values <10%. Additionally, the layers between 10 and 30 cm, between 30 and 50 cm and between 50 and 70 cm also demonstrated notable dryness, with ~13%, 22% and 18% REW, respectively. Following irrigation of the CO plots on D0, an increase in REW was observed, followed by a gradual and consistent decline in accordance with the typical progression of the growing season. A notable increase in REW was observed in the TE plots, reaching a plus of between 10% and 50% across all layers from D0 to D4. However, the 0–7 cm depth exhibited the least pronounced increase. From D3, a slight decrease was observed as the trees absorbed the irrigation water, until from D42, a steady increase in REW was observed for all layers due to the opened roofs and rainfall events. At D63, the CO and TE plots exhibited similar REW values (Figure 1a). The SWC data can be found in Figure S1 available as Supplementary Data at *Tree Physiology* Online.

Sap flow density (SF) of CO and TE beech did not change during the watering and remained constant over the whole period with CO (11.1 ± 4.2 l dm^−2^ day^−1^) being slightly higher than TE (8.1 ± 2.2 l dm^−2^ day^−1^) by ~25% (Table 3). In spruce, only CO (6.9 ± 3.4 l dm^−2^ day^−1^) showed constant SF over the whole measurement period, while in TE, there was a slight increase of ~11% after the onset of watering (before watering: 3.5 ± 2.7 l dm^−2^ day^−1^ and after watering: 3.9 ± 2.7 l dm^−2^ day^−1^). Overall SF of TE spruce was reduced by about 40–50% compared with CO (Table 3).

**Table 3:** Leaf water content (LWC in %), absolute (EER in ‰) and relative to the source water (ΔLW) evaporative enrichment of leaf water and deuterium signal of twig water (δ^2^H_twig_ in ‰) of beech and spruce under control (CO) and throughfall exclusion (TE). Values are given as the mean ± 1 SD.

SF (l dm^−2^ day^−1^)	Beech		Spruce	
	CO	TE	CO	TE
pre (Day −7 to −1)	11.2 ± 4.2	8.1 ± 2.8	6.8 ± 2.9	3.5 ± 2.7
D0	12.4 ± 4.1	8.7 ± 1.0	7.0 ± 3.3	3.0 ± 1.8
D1	11.3 ± 4.3	9.4 ± 2.0	6.9 ± 3.3	3.9 ± 2.9
D2	11.0 ± 4.3	9.4 ± 2.0	6.3 ± 3.2	3.8 ± 2.7
D3	8.7 ± 5.0	5.6 ± 3.0	6.6 ± 4.2	3.4 ± 2.3
D4	10.0 ± 3.3	7.3 ± 2.2	6.8 ± 3.3	3.9 ± 2.7
D5	12.1 ± 3.8	9.3 ± 2.7	7.8 ± 3.5	4.5 ± 2.8
D6	11.3 ± 3.7	8.3 ± 1.5	6.4 ± 3.0	4.2 ± 3.3
D7	11.6 ± 4.5	7.0 ± 3.0	7.2 ± 3.7	4.0 ± 3.0
Post (Day 8–15)	11.6 ± 4.6	7.6 ± 2.1	7.2 ± 3.4	4.1 ± 2.7

### Effect of drought on the root water uptake depth of beech and spruce

Before irrigation, the linear regression between δ^2^H_soil_ and the logarithmic soil depth (Figure S2 available as Supplementary Data at *Tree Physiology* Online) was significantly different between CO and TE (*P*-value < 0.001), with high *R*^2^ values for CO (0.91) and TE (0.92).

In both species, the mean RWU on the CO plots was close to 30 cm depth (Table 1). On the TE plots, the RWU was significantly shifted upwards in both species (*P*-value < 0.01, Table 1), with beech being slightly shallower (10.9 ± 7.6 cm) than spruce (15.9 ± 4.3 cm).

### Tracing of irrigation water along the soil–plant continuum

#### Before irrigation

**Figure 2:**
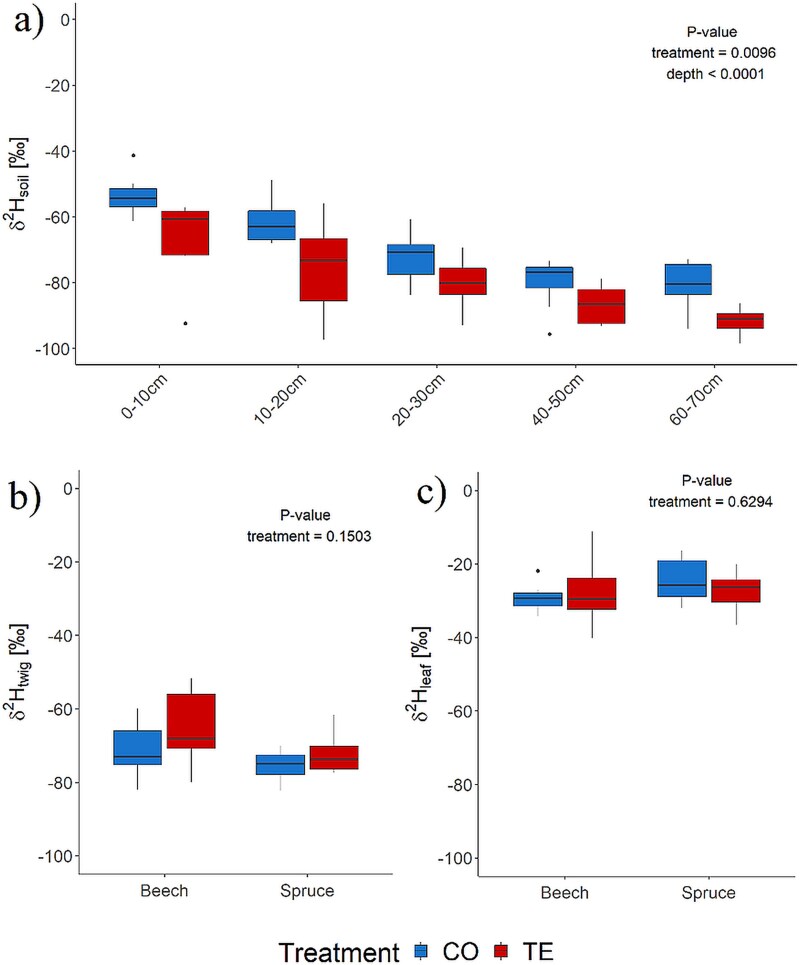
Initial deuterium signature (δ^2^H in ‰) on D−6 before the irrigation of soil (a), twig (b) and leaf water (c) for beech and spruce under control (CO—blue) and throughfall exclusion (TE—red).

Depth and TE significantly affected δ^2^H_soil_ (*P*-value < 0.05), with δ^2^H_soil_ decreasing with depth and δ^2^H_soil_ being generally more enriched in CO than TE (Figure 2). δ^2^H_soil_ of CO soils ranged from −53.3 ± 5.7‰ in the shallowest layer (0–10 cm) to −81.2 ± 7.8‰ in the deepest layer (60–70 cm), while on average, ${\delta^2{\textrm{H}}}$ of TE soil was 10.8 ± 3.2‰ lower (top layer: −66.7 ± 11.6‰ and deepest layer: −91.6 ± 3.9‰, Figure 2a).

Before irrigation, no significant differences between CO vs. TE and beech vs. spruce were found for δ^2^H_twig_ and δ^2^H_leaf_. δ^2^H_twig_ on D−6 in beech was at −71.2 ± 8.1 ‰ (CO) and −65.2 ± 11.3 ‰ (TE) and for spruce −75.5 ± 4.5 ‰ (CO) and − 72.1 ± 6.0 ‰ (TE), respectively (Figure 2b). For δ^2^H_leaf_, no differences were found between treatments and measurement days (D−6 and D−1), but beech trees showed slightly lower values (mean of D−6 and D−1: CO: −29.5 ± 3.5‰ and TE: −28.1 ± 7.5‰) than spruce (mean of D−6 and D−1: CO: −24.5 ± 5.8‰ and TE: −27.5 ± 5.4 ‰, Figure 2c).

#### Changes in the isotopic signature upon irrigation with ^2^H-enriched water

Upon irrigation, δ^2^H_soil_ on CO plots, especially in the three uppermost layers (0–30 cm), increased (Δδ^2^H_soil_) on the day of the irrigation by 180–350‰ (Figure 1b). However, Δδ^2^H_soil_ in lower depths of CO plots was lower, for 40–50 cm on D0 at ~45‰ and to a maximum of plus 120‰ on D4–D7 and for 60–70 cm constantly at 45‰ from D0 onward. A more homogenous distribution of the irrigation water across soil depths was observed on TE plots, which could be linked to the amount of irrigation water (for more information, see Discussion). Δδ^2^H_soil_ rose during the first 4 days in all depths to a maximum of 150–200‰ over all five depths (Figure 1b).

**Figure 3:**
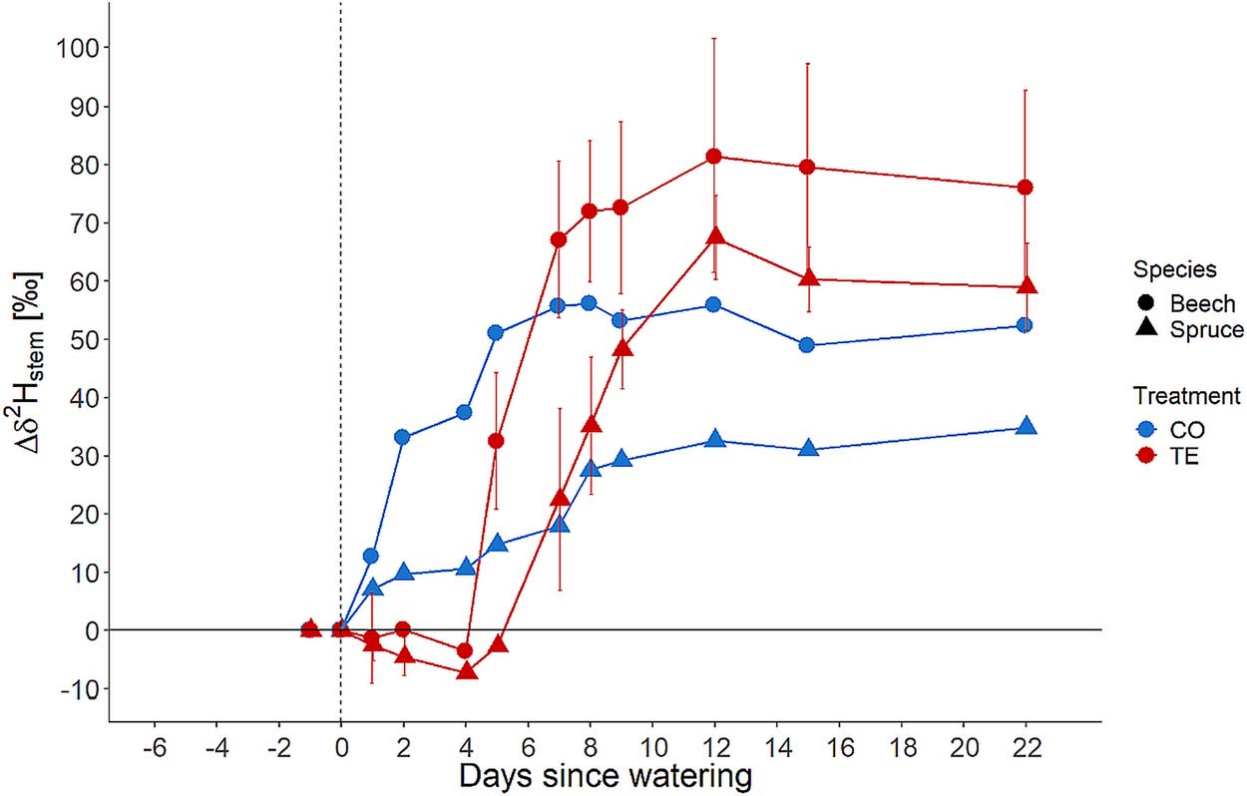
Deuterium excess (Δδ^2^H) of stem sapwood water for beech (circle) and spruce (triangle) in CO (blue) and TE (red) plots. Significant increase in CO beech and spruce on D1, TE beech on D5 and TE spruce on D7. The vertical dashed line marks the irrigation on D0. Symbols show the mean ± 1 SD.

In stems at DBH of CO trees of both species, ^2^H-labeled water was detected several days earlier than in the sapwood of TE trees. In CO trees, as early as ~ 7 h upon irrigation, an increase (Δδ^2^H_stem_) of ~10‰ was detected in stems at DBH and raised over the following 3 weeks to a Δδ^2^H_stem_ of up to 55‰ and 35‰ in beech and spruce, respectively. Conversely, the detection of labeled water in stems of TE trees was significantly delayed, i.e., on D5 for beech and D7 for spruce (Figure 3). The maximum Δδ^2^H_stem_ in stem water of TE trees was 80‰ and 60‰ for beech and spruce, respectively. A significant increase was found on D15 for beech and spruce in the twig water, but no differences were found between CO and TE (Table 2).

**Figure 4:**
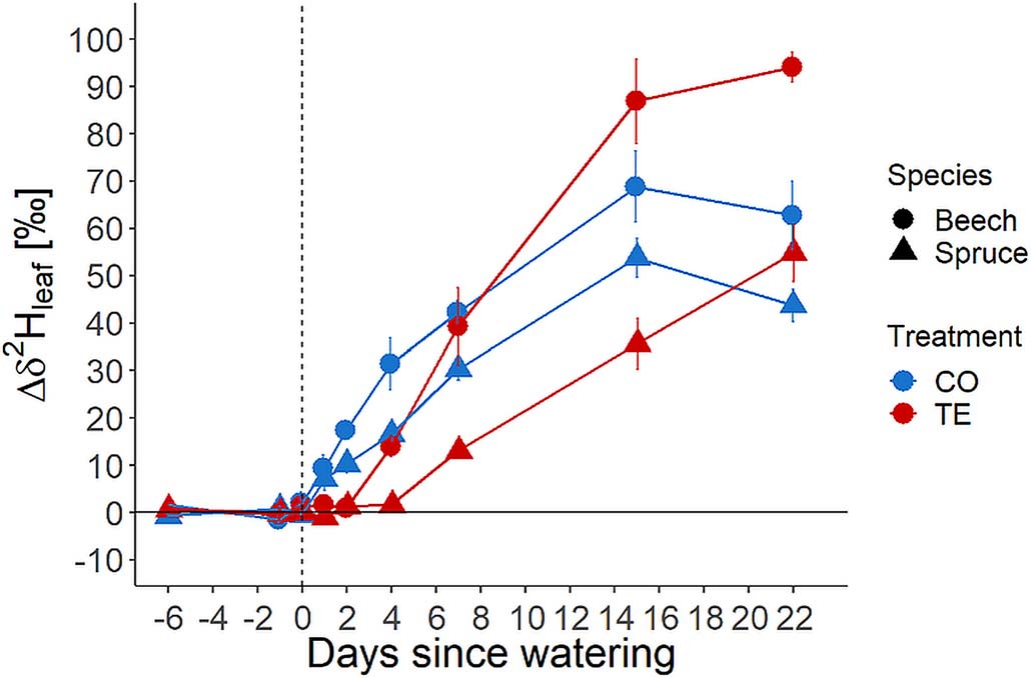
Deuterium excess (Δδ^2^H) of leaf water for beech (circle) and spruce (triangle) and CO (blue) and TE (red). Significant increase in CO beech and spruce on D1, TE beech on D4 and TE spruce on D7. The vertical dashed line marks the irrigation on D0. Symbols show the mean ± 1 SD.

The dynamics in the Δδ^2^H_leaf_ were similar to Δδ^2^H_stem_. In CO trees already on D1 after labeling, a significant increase was found in the leaf water for both species (beech: 10‰ and spruce: 7‰, Figure 4). In TE trees, this was significantly delayed for both species by several days. For beech on D4, a significant increase of Δδ^2^H_leaf_ was found (Δδ^2^H_leaf_ of 14‰) and for spruce, only on D7 (Δδ^2^H_leaf_ of 13‰). After 15 days, both treatments showed similar Δδ^2^H_leaf_, with values of 65–85‰ in beech and 35–55‰ in spruce (Figure 4). Treatment (CO vs TE) had no effect on LWC for both species, and also, irrigation did not change LWC (Table 2).

### Turnover of water pools along the SPC

**Figure 5:**
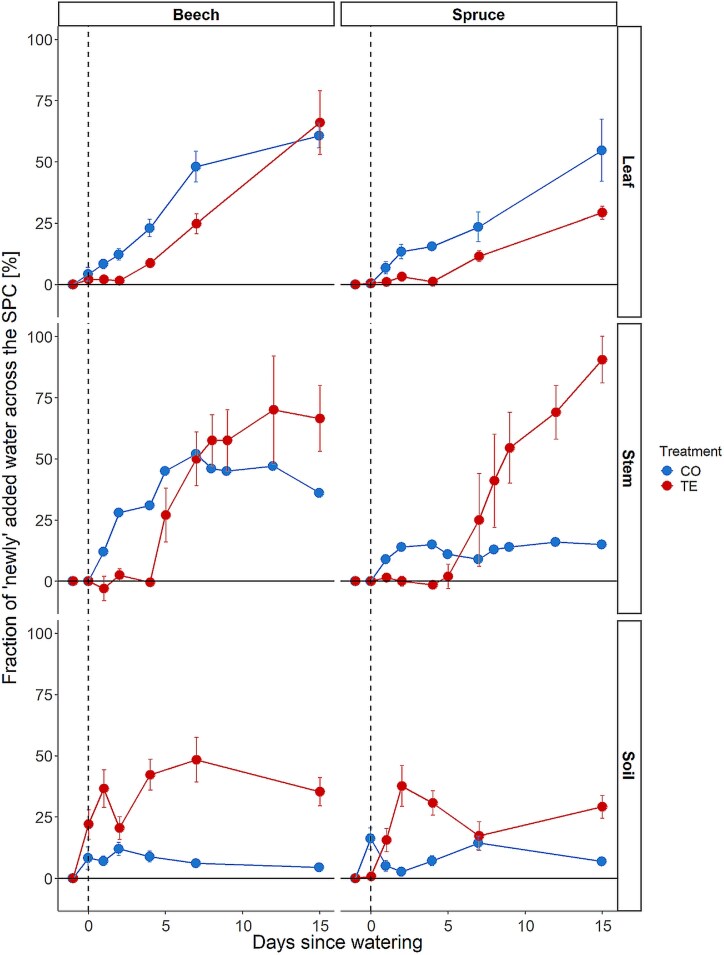
Fractions of irrigation water [%] after the irrigation in different compartments of the SPC for beech and spruce under control (CO, blue) and throughfall exclusion (TE, red) treatment. Symbols show the mean ± 1SE.

In the soil water of CO plots, only a minor fraction consisted of labeled water. However, the fraction of irrigation water remained relatively constant over 2 weeks at 8 ± 4% (Figure 5). For the TE plots, a significant fraction of the water found after the labeling was irrigation water (28 ± 13%, Figure 5). Irrigation water was rather homogenously distributed across the different soil depths.

A similar pattern was found in the stem sapwood water, with CO trees only showing a small fraction of irrigation water (average of 38% in beech and 13% in spruce over time), which was constant from D2 onward (Figure 5). In TE trees, the fraction of labeled water rose on average to 58% in beech from D5 and 62% in spruce from D7 on. The level in TE beech remained relatively constant from D7/8, while in TE spruce, it increased constantly to >80% until D15 (Figure 5).

The leaf’s fraction of labeled water increased differently from the stem and soil. A constant rise was found over 2 weeks for both species and treatments (Figure 5). For CO trees already on D1, a significant fraction of irrigation water was found in beech (8%) and spruce (7%). By D15, this proportion increased to 61% in beech and 55% in spruce in the CO treatment (Figure 5). The delay of 4 days for beech and 7 days for spruce in the arrival of irrigation water, as observed in the sapwood, was also identified in the leaf water. Levels of irrigation water in beech leaves rose to 9% on D4 and 66% on D15 and in spruce to 12% on D7 and 30% on D15 (Figure 5).

## Discussion

This study aimed to estimate the water uptake and intra-tree distribution of previously drought-stressed mature beech and spruce by δ^2^H-labeling during the first days of drought recovery. The δ^2^H-signature of soil, stem and leaf water was assessed before and during the first 2 weeks after drought release by controlled irrigation (Grams et al. 2021). The results showed that there was no difference in tracer uptake between well-watered control trees of both species. However, both species experienced delayed water uptake during drought recovery compared with control trees. Furthermore, recovering spruce trees demonstrated a significantly slower uptake and distribution of newly available water compared with recovering beech trees.

### Effects of drought on the mean water uptake depth assessed by soil/stem water isotopic signature

The lower soil water isotopic signature in TE compared with CO soil seems counterintuitive at first (Figure 2a), as it is known from the literature that drought treatments increase the isotopic signature of soil surface water through evaporation (Dawson and Ehleringer 1998). In the present experiment, only the summer precipitation was excluded, so the precipitation’s δ^2^H-signature and its change over the season must also be considered (Dansgaard 1964). According to the model of Bowen et al. (2005), the δ^2^H-signature of the winter precipitation (November to February) at the Kroof site, when all plots receive precipitation, is about −94.3 ± 8.7 ‰, while the summer precipitation (roofs closed and therefore excluded rainfall on the TE plots, March to October) is much higher: −51.8 ± 20.2‰ (annual mean: 65.9 ± 26.8‰). As TE plots only received the winter precipitation, the δ^2^H of soil water must be lower than the signature of CO, with evaporative enrichment of soil surface water potentially counteracting this only to a minor extent.

Given that TE trees, on average, take up more water from shallower layers than CO trees according to our findings (Table 1 and Figure S2 available as Supplementary Data at *Tree Physiology* Online), the similar δ^2^H values of twig and leaf water δ^2^H-signatures appear to be a plausible outcome. This is because the δ^2^H-signature of the CO plots is slightly higher at all measured soil depths than in TE plots (Figure 2a). However, the shift in RWU to shallower layers in the drought treatment was unexpected and contradicts recent literature (Bachofen et al. 2024) and our hypothesis H1, which postulates that TE trees shift their mean RWU to deeper soil layers during drought years. One potential factor influencing the calculation of RWU is the reduction in xylem water transport resulting from drought conditions. This phenomenon was more pronounced in spruce than in beech (Table 2, Hesse et al. 2023). Given the substantial difference between beech and spruce, it is evident that the potential enrichment of xylem water during transport via exchange with the phloem sap or other biochemical reactions (Martín-Gómez et al. 2017, Montemagno et al. 2023) cannot fully account for the observed shift in water uptake depth. Nevertheless, similar results have been demonstrated in a Swiss forest stand for spruce, with no alterations in water uptake depth under fluctuating water availability and only minor (Brinkmann et al. 2019, Hackmann et al. 2025) to no changes for beech (Gessler et al. 2022) and for herbaceous species (Prechsl et al. 2015). Furthermore, the limited water availability throughout the entire soil depth (up to 70 cm), as indicated by the plant-available water data from the same experimental site (Figure 1a, Grams et al. 2021), may have compelled the plants to develop roots closer to the surface in order to receive the winter precipitation as soon as possible. This is particularly evident given that both species lost a considerable number of vital root tips during the drought treatment (Nickel et al. 2018). Therefore, we reject H1 as both species moved their RWU to shallower layers, with beech being even shallower than spruce. We furthermore assumed that RWU would not change within the first 15 days after the watering, as the major amount of newly assimilated carbon was transported belowground, at least for spruce, into the shallow soil layer (0–30 cm, Hikino et al. 2022*a*, 2022*b*). Additionally, Zang et al. (2014) reported limited root growth after drought stress in juvenile beech trees and Olesinski et al. (2011) found that full recovery in balsam fir only occurred in the next growing season.

### Distribution of labeled irrigation water during the first days of drought recovery along the soil–plant continuum

δ^2^H-enriched water added by irrigation was found in all parts of the SPC, i.e., soil, stem water and leaves, for CO and TE plots (Figures 1b, 3 and 4). However, the time of detection and distribution varied depending on the drought treatment and species. The δ^2^H-signal was detectable after ~24 h in the soil of the CO and TE plots in all depths (Figure 1b). The label distribution in the soils of the TE plots was relatively homogeneous (see the following paragraph for a detailed discussion). In contrast, the soils of the CO plots exhibited a higher δ^2^H-signature in the shallow soil layers throughout the experimental period. This finding is consistent with other studies that have demonstrated that water from deeper soil layers is only replaced or mixed by heavy rainfall or snowmelt (Gazis and Feng 2004, Brinkmann et al. 2018), but the different amounts of water added during irrigation on CO and TE plots should also be considered and might be one reason for the more homogenous distribution across the TE plots.

Within both tree species, the δ^2^H-signature of the irrigation water was found earlier in CO trees, i.e., on D1 after labeling, than in TE trees (Figures 3 and 4). Irrigation water detection was delayed by 3 (TE beech) to 6 (TE spruce) days compared with CO trees. Labeled water was detected ~3 days earlier in TE beech than in TE spruce in both stem sapwood xylem and leaves (Figures 3 and 4). We therefore reject hypothesis H2a, which stated that trees under TE would take up irrigation water on the day of irrigation, with shallower-rooted spruce having access to irrigation water earlier than deeper-rooted beech. The leaf water of TE beech showed an increase in δ^2^H at D4 and TE spruce at D7, whereas in CO trees of both species, δ2H showed increased values at D1 (Figure 4). Thus, we accepted H2b, i.e., trees under TE absorbed irrigation water and distributed it within the tree to the stem xylem and leaves faster in beech than in spruce. The discrepancy in distribution times between recovering trees (TE) and CO trees may be attributed to the replenishment of water reserves within the coarse root and stem xylem structures. It has been postulated that mature trees possess a considerable capacity for water storage within xylem tissues (Holbrook 1995, Čermák et al. 2007, Matheny et al. 2015, Preisler et al. 2022). During the drought periods, these internal storages have been reduced, particularly in spruce (Knüver et al. 2022), and were potentially refilled (Betsch et al. 2011) in the course of the irrigation as suggested by Hesse et al. (2023) for trees of the same experiment. Another possibility that could explain this delay would be the loss of soil–root contact on the TE plots. Under drought stress, the soil shrinks, creating an air-filled space around the roots. This would significantly decrease water uptake as the thin air layer drastically reduces water conductivity between the soil and roots (Carminati et al. 2009, Delval et al. 2025). The recovery of this soil–root contact is hardly studied and is not yet quantified (Zheng et al. 2023) An additional explanation is impaired xylem water transport, as trees subjected to drought conditions tend to exhibit reduced water transport, which can potentially lead to tree death (Geßler et al. 2007, Mantova et al. 2022, McDowell et al. 2022). Such a reduction was observed for the xylem sap flow density in beech and, to a greater extent, in spruce for the same experimental trees (Table 3, Hesse et al. 2023, 2024). The different hydraulic architecture (e.g., lower hydraulic conductivity) of conifers in comparison with angiosperms (Brodribb et al. 2005, Johnson et al. 2012) could also partially explain the time difference of 3 days in leaf δ^2^H-signature enrichment between TE beech and TE spruce. Nevertheless, a comparable discrepancy between CO beech and CO spruce should have been observed if hydraulic architecture was the main determining factor, but both trees showed enriched stem and leaf water already on D1 under control conditions (Figures 3 and 4). Thus, the xylem sap flow alone is an insufficient explanation for the observed differences; for a more detailed discussion about the sap flow during the recovery, please refer to Hesse et al. (2023). Other potential explanations are the significant loss of fine roots in TE spruce and TE beech at the experimental site (Nickel et al. 2018), rhizosphere hydrophobicity (Zarebanadkouki and Carminati 2014, Zarebanadkouki et al. 2016) or a reduction of root-surface contact (North and Nobel 1997, Carminati et al. 2009) with negative consequences for the water uptake capacity (Tschaplinski and Blake 1985). Additionally, spruce suberizes its fine roots during periods of severe drought, thereby further diminishing its water uptake ability (Nikolova et al. 2020). The depth of RWU did not appear to be a significant factor in water uptake timing and patterns following irrigation, as both species utilize water from similar soil layers (Table 1). Most likely, the delay in water uptake between CO and TE trees can be attributed to a combination of soil properties, such as soil hydrophobicity and loss of soil–root contact, and additional plant-related factors, such as refilling of internal water storage and reducing sap flow in TE trees. However, the difference between TE beech and TE spruce is probably mainly caused by plant-related properties, such as their different responses to the previous drought, with spruce generally responding more strongly than beech. For example, strongly reduced sap flow, tightly controlled stomatal opening, and significant loss of internal water storage of spruce under recurrent drought (Tomasella et al. 2018, Pretzsch et al. 2020, Knüver et al. 2022, Hesse et al. 2023, 2024).

### Soil and tree water pool turnover upon irrigation

The distribution of deuterated water after irrigation was generally very different between CO and TE in both soil and tree compartments. The high proportion of irrigation water in the soil of the TE plots compared with the low proportion on the CO plots (Figure 5, bottom) can be attributed to the different amounts of water added and the very low initial water content on the TE plots. However, an evident pattern emerged on the CO plots, indicating that the shallower soil layers contained a greater proportion of irrigation water than the deeper soil layers (Figure 1b). This indicates that the water initially mixed in the upper layer and then gradually infiltrated deeper, becoming increasingly diluted with depth (Gisi 1997, Huang et al. 2013). In TE, the pattern was more variable, as the irrigation water was potentially moving directly to different depths rather than percolating through them. The highly hydrophobic nature of the soil, resulting from 5 years of TE of summer precipitation, led to a significant reduction in water infiltration (see Grams et al. 2021). Given that this hydrophobicity had to be overcome before water could infiltrate the shallowest soil, a process that can take several days (Burch et al. 1989, Bauters et al. 2000), it is plausible that some parts of the water may have taken different routes, driven by the hydrophobicity of the topsoil (Gimbel et al. 2016). One potential explanation is the formation of soil cracks, which can occur naturally as a consequence of severe drought (Novàk et al. 2000). Alternatively, the presence of root and animal pipes may also be a contributing factor (Amelung et al. 2018), as these have been observed on the experimental sites on several occasions (personal observations).

The δ^2^H of the stem sapwood xylem water was quickly changed and subsequently stabilized in CO trees after irrigation for both species (Figure 5, middle). The irrigation water was, therefore, probably not directly transported upward to the leaves, but mixing with stored water in the stem did occur to some extent. Studies for other species report that up to 50% of transpired water originates from stem water storage (Waring et al. 1979, Hao et al. 2013, Liu et al. 2021, Poyatos et al. 2021). A comparable pattern was observed in the TE trees, with an even more pronounced increase in the δ^2^H-signature of the sapwood water (and a plateau in beech only) but with a time delay of several days in both species (Figure 5, middle). This delay could indicate that within several days or weeks, the water storage in the inner xylem of the trees will be replenished (i.e., stable level of newly added water in Figure 5, middle), with beech again showing a faster response (~7 days) than spruce (at least 15 days as no flattening of the curve was observed until D15 in Figure 5, middle). Additionally, this also indicates a mixing of freshly absorbed water with water stored in the inner xylem before transporting it upward to the leaves (Anderegg et al. 2012, Liu et al. 2021). Despite the aforementioned time lag in TE trees between beech and spruce, a similar increase in the fraction of irrigation water was found in the leaves for both species (Figure 5, top). The fraction of labeled water increased continuously over the experimental period to the same level for CO and TE in beech and spruce, respectively. These findings indicate that leaves have a high priority in water allocation upon drought stress release, despite the mixing of newly absorbed and stored water in the xylem, which is also reflected in the very quick recovery of leaf physiology upon drought release (Ruehr et al. 2019, Hesse et al. 2023, Wagner et al. 2023). Additionally, our results suggest that leaves have minimal capacity for internal water storage, as the ^2^H values of the leaf continuously increased without reaching a plateau. Therefore, we accept H3, that water pools in trees, i.e., stem and leaves, and soil under previous TE have a faster turnover time compared with unstressed trees and soils, for the soil water pool. However, we reject it for the tree organs, as TE trees showed a slower water pool turnover compared with CO. However, TE trees showed much higher fractions of irrigation water in the stem and soil water pool (except for leaves). This could be connected to the lower water content in drought-stressed trees (Konings et al. 2021) and the re-filling of internal water storage (Hesse et al. 2023, Martín-Gómez et al. 2023).

## Conclusions

Our findings highlight species-specific differences in drought recovery, with beech exhibiting a faster distribution of newly available water compared with spruce. The delayed uptake and distribution in drought-stressed trees, particularly in spruce, suggests that stem water refilling might play a critical role in re-establishing hydraulic function before soil water can be effectively utilized. Additionally, the shift in water uptake toward shallower soil layers indicates the importance of surface moisture availability in post-drought recovery. Further research should explore the long-term consequences of repeated drought events on tree hydraulic function, soil–root interaction and tree internal water storage. Understanding how trees balance stem water refilling with transpiration demands will be essential for predicting future forest resilience. Additionally, investigating the role of soil properties, microbial activity and mycorrhizal associations in water uptake under drought and rewetting conditions could provide deeper insights into belowground recovery mechanisms. Advanced techniques, such as high-resolution stable isotope tracing and non-invasive imaging of root dynamics, could further refine our understanding of post-drought water distribution and uptake.

## Supplementary Material

tpaf153_Hesse_KROOF_deuterium_labeling_supplement

## Data Availability

Data will be made available upon reasonable request.

## Notes

Handling Editor: Dr. Roberto Tognetti

## References

[ref1] Amelung W, Blume H-P, Fleige H et al. (2018) Lehrbuch der Bodenkunde, 17th edn. Berlin, Springer Verlag.

[ref2] Anderegg WRL, Berry JA, Field CB. (2012) Linking definitions, mechanisms, and modeling of drought-induced tree death. Trends Plant Sci 17:693–700. 10.1016/j.tplants.2012.09.006. 23099222

[ref3] Andivia E, Madrigal-González J, Villar-Salvador P, Zavala MA. (2018) Do adult trees increase conspecific juvenile resilience to recurrent droughts? Implications for forest regeneration. Ecosphere 9:1–13. 10.1002/ecs2.2282.

[ref4] Bachofen C, Tumber-Dávila SJ, Mackay DS, McDowell NG, Carminati A, Klein T, Stocker BD, Mencuccini M, Grossiord C. (2024) Tree water uptake patterns across the globe. New Phytol 242:1891–1910. 10.1111/nph.19762. 38649790

[ref5] Bauters TWJ, Steenhuis TS, Dicarlo DA et al. (2000) Physics of water repellent soils. J Hydrol 231-232:233–243. 10.1016/S0022-1694(00)00197-9.

[ref6] Betsch P, Bonal D, Breda N et al. (2011) Drought effects on water relations in beech: the contribution of exchangeable water reservoirs. Agric For Meteorol 151:531–543. 10.1016/j.agrformet.2010.12.008.

[ref7] Børja I, Godbold DL, Svetlik J et al. (2017) Norway spruce fine roots and fungal hyphae grow deeper in forest soils after extended drought. In: Martin Lukac, Paola Grenni, Mauro Gamboni (eds), Soil biological communities and ecosystem resilience. Cham, Springer International Publishing, pp 123–142. 10.1007/978-3-319-63336-7_8.

[ref8] Bowen GJ, Wassenaar LI, Hobson KA. (2005) Global application of stable hydrogen and oxygen isotopes to wildlife forensics. Oecologia 143:337–348. 10.1007/s00442-004-1813-y. 15726429

[ref9] Brinkmann N, Eugster W, Buchmann N, Kahmen A. (2019) Species-specific differences in water uptake depth of mature temperate trees vary with water availability in the soil. Plant Biol 21:71–81. 10.1111/plb.12907. 30184305

[ref10] Brinkmann N, Seeger S, Weiler M, Buchmann N, Eugster W, Kahmen A. (2018) Employing stable isotopes to determine the residence times of soil water and the temporal origin of water taken up by *Fagus sylvatica* and *Picea abies* in a temperate forest. New Phytol 219:1300–1313. 10.1111/nph.15255. 29888480

[ref11] Brodribb TJ, Bowman DJMS, Nichols S, Delzon S, Burlett R. (2010) Xylem function and growth rate interact to determine recovery rates after exposure to extreme water deficit. New Phytol 188:533–542. 10.1111/j.1469-8137.2010.03393.x. 20673281

[ref12] Brodribb TJ, Holbrook NM, Hill RS. (2005) Seedling growth in conifers and angiosperms: impacts of contrasting xylem structure. Aust J Bot 53:749–755. 10.1071/BT05049.

[ref13] Burch GJ, Moore ID, Burns J. (1989) Soil hydrophobic effects on infiltration and catchment runoff. Hydrol Process 3:211–222. 10.1002/hyp.3360030302.

[ref14] Carminati A, Vetterlein D, Weller U, Vogel HJ, Oswald SE. (2009) When roots lose contact. Vadose Zo J 8:805–809. 10.2136/vzj2008.0147.

[ref15] Cavender-Bares J, Bazzaz FA. (2000) Changes in drought response strategies with ontogeny in *Quercus rubra*: implications for scaling from seedlings to mature trees. Oecologia 124:8–18. 10.1007/PL00008865. 28308415

[ref16] Cavin L, Mountford EP, Peterken GF, Jump AS. (2013) Extreme drought alters competitive dominance within and between tree species in a mixed forest stand. Funct Ecol 27:1424–1435. 10.1111/1365-2435.12126.

[ref17] Čermák J, Kučera J, Bauerle WL et al. (2007) Tree water storage and its diurnal dynamics related to sap flow and changes in stem volume in old-growth Douglas-fir trees. Tree Physiol 27:181–198. 10.1093/treephys/27.2.181. 17241961

[ref18] Cernusak LA, Barbour MM, Arndt SK et al. (2016) Stable isotopes in leaf water of terrestrial plants. Plant Cell Environ 39:1087–1102. 10.1111/pce.12703. 26715126

[ref19] Dansgaard W . (1964) Stable isotopes in precipitation. Tellus 16:436–468. 10.3402/tellusa.v16i4.8993.

[ref20] Dawson TE, Ehleringer JR. (1998) Plants, isotopes and water use: a catchment-scale perspective. In: Carol Kendall, Jeffrey J. McDonnell (eds), Isotope tracers in catchment hydrology. Amsterdam, Elsevier, pp 165–202. 10.1016/B978-0-444-81546-0.50013-6.

[ref21] Delval L, Vanderborght J, Javaux M. (2025) Combination of plant and soil water potential monitoring and modelling demonstrates soil-root hydraulic disconnection during drought. Plant Soil 511:1449–1472. 10.1007/s11104-024-07062-2.

[ref22] Englund G, Cooper SD. (2003) Scale effects and extrapolation in ecological experiments. Adv Ecol Res 33:161–213. 10.1016/s0065-2504(03)33011-9.

[ref23] Farquhar GD, Cernusak LA. (2005) On the isotopic composition of leaf water in the non-steady state. Funct Plant Biol 32:293–303. 10.1071/FP04232. 32689132

[ref24] Gallé A, Haldimann P, Feller U. (2007) Photosynthetic performance and water relations in young pubescent oak (*Quercus pubescens*) trees during drought stress and recovery. New Phytol 174:799–810. 10.1111/j.1469-8137.2007.02047.x. 17504463

[ref25] Gazis C, Feng X. (2004) A stable isotope study of soil water: evidence for mixing and preferential flow paths. Geoderma 119:97–111. 10.1016/S0016-7061(03)00243-X.

[ref26] Gebhardt T, Hesse BD, Hikino K, Kolovrat K, Hafner BD, Grams TEE, Häberle KH. (2023) Repeated summer drought changes the radial xylem sap flow profile in mature Norway spruce but not in European beech. Agric For Meteorol 329:109285. 10.1016/j.agrformet.2022.109285.

[ref27] Gessler A, Bächli L, Rouholahnejad Freund E et al. (2022) Drought reduces water uptake in beech from the drying topsoil, but no compensatory uptake occurs from deeper soil layers. New Phytol 233:194–206. 10.1111/nph.17767. 34610146 PMC9293437

[ref28] Geßler A, Keitel C, Kreuzwieser J, Matyssek R, Seiler W, Rennenberg H. (2007) Potential risks for European beech (*Fagus sylvatica* L.) in a changing climate. Trees Struct Funct 21:1–11. 10.1007/s00468-006-0107-x.

[ref29] Gimbel KF, Puhlmann H, Weiler M. (2016) Does drought alter hydrological functions in forest soils? Hydrol Earth Syst Sci 20:1301–1317. 10.5194/hess-20-1301-2016.

[ref30] Gisi U . (1997) Bodenökologie, 2nd edn. Stuttgart, Georg Thieme Verlag.

[ref31] Goisser M, Geppert U, Rötzer T et al. (2016) Does belowground interaction with *Fagus sylvatica* increase drought susceptibility of photosynthesis and stem growth in *Picea abies*? For Ecol Manag 375:268–278. 10.1016/j.foreco.2016.05.032.

[ref32] Grams TEE, Hesse BD, Gebhardt T et al. (2021) The Kroof experiment: realization and efficacy of a recurrent drought experiment plus recovery in a beech/spruce forest. Ecosphere 12:1–20. 10.1002/ecs2.3399.

[ref33] Granier A . (1987) Evaluation of transpiration in a Douglas-fir stand by means of sap flow measurements. Tree Physiol 3:309–320. 10.1093/treephys/3.4.309. 14975915

[ref34] Groover A, Holbrook NM, Polle A et al. (2025) Tree drought physiology: critical research questions and strategies for mitigating climate change effects on forests. New Phytol 245:1817–1832. 10.1111/nph.20326. 39690524

[ref35] Hackmann CA, Paligi SS, Mund M, Hölscher D, Leuschner C, Pietig K, Ammer C. (2025) Root water uptake depth in temperate forest trees: species-specific patterns shaped by neighbourhood and environment. Plant Biol Early View 10.1111/plb.70058. 40523115

[ref36] Hafner BD, Tomasella M, Häberle K et al. (2017) Hydraulic redistribution under moderate drought among English oak, European beech and Norway spruce determined by deuterium isotope labeling in a split-root experiment. Tree Physiol 63:242–248. 10.1093/tropej/fmw080. 28541559

[ref37] Hafner BD, Hesse BD, Bauerle TL, Grams TEE. (2020) Water potential gradient, root conduit size and root xylem hydraulic conductivity determine the extent of hydraulic redistribution in temperate trees. Funct Ecol 34:561–574. 10.1111/1365-2435.13508.

[ref38] Hao GY, James KW, Holbrook NM, Goldstein G. (2013) Investigating xylem embolism formation, refilling and water storage in tree trunks using frequency domain reflectometry. J Exp Bot 64:2321–2332. 10.1093/jxb/ert090. 23585669 PMC3654422

[ref40] Hartmann H, Ziegler W, Kolle O, Trumbore S. (2013) Thirst beats hunger - declining hydration during drought prevents carbon starvation in Norway spruce saplings. New Phytol 200:340–349. 10.1111/nph.12331. 23692181

[ref39] Hartmann H, Moura CF, Anderegg WRL et al. (2018) Research frontiers for improving our understanding of drought-induced tree and forest mortality. New Phytol 218:15–28. 10.1111/nph.15048. 29488280

[ref42] Hesse BD, Goisser M, Hartmann H, Grams TEE. (2019) Repeated summer drought delays sugar export from the leaf and impairs phloem transport in mature beech. Tree Physiol 39:192–200. 10.1093/treephys/tpy122. 30388272

[ref43] Hesse BD, Hartmann H, Rötzer T, Landhäusser SM, Goisser M, Weikl F, Pritsch K, Grams TEE. (2021) Mature beech and spruce trees under drought – higher C investment in reproduction at the expense of whole-tree NSC stores. Environ Exp Bot 191:104615. 10.1016/j.envexpbot.2021.104615.

[ref41] Hesse BD, Gebhardt T, Hafner BD, Hikino K, Reitsam A, Gigl M, Dawid C, Häberle KH, Grams TEE. (2023) Physiological recovery of tree water relations upon drought release – response of mature beech and spruce after five years of recurrent summer drought. Tree Physiol 43:522–538. 10.1093/treephys/tpac135. 36413114

[ref44] Hesse BD, Hikino K, Gebhardt T, Buchhart C, Dervishi V, Goisser M, Pretzsch H, Häberle KH, Grams TEE. (2024) Acclimation of mature spruce and beech to five years of repeated summer drought – the role of stomatal conductance and leaf area adjustment for water use. Sci Total Environ 951:175805. 10.1016/j.scitotenv.2024.175805. 39197757

[ref45] Hikino K, Danzberger J, Riedel V et al. (2022 *a*) High resilience of carbon transport in long-term drought-stressed mature Norway trees within 2 weeks after drought release. Glob Chang Biol 28:2095–2110. 10.1111/gcb.16051. 34927319

[ref46] Hikino K, Danzberger J, Riedel VP et al. (2022 *b*) Dynamics of initial C allocation after drought release in mature Norway spruce - increased belowground allocation of current photoassimilates covers only half of the C used for fine-root growth. Glob Chang Biol 28:6889–6905. 10.1111/gcb.16388. 36039835

[ref47] Holbrook NM . (1995) Stem water storage. In: Barbara L Gartner (ed), Plant stems: physiology and functional morphology. Academic Press, San Diego, CA, pp 151–174. 10.1016/B978-012276460-8/50009-6.

[ref48] Huang J, Wu P, Zhao X. (2013) Effects of rainfall intensity, underlying surface and slope gradient on soil infiltration under simulated rainfall experiments. Catena 104:93–102. 10.1016/j.catena.2012.10.013.

[ref49] IAEA . (2017) Reference sheet for international measurement standards VSMOW2, SLAP2. International Atomic Energy Agency. https://nucleus.iaea.org/sites/AnalyticalReferenceMaterials/Shared%20Documents/ReferenceMaterials/StableIsotopes/VSMOW2/VSMOW2_SLAP2.pdf

[ref50] IPCC . (2014) Contribution of working groups I, II and III to the fifth assessment report of the Intergovernmental Panel on Climate Change. In: Pachauri RK, Meyer LA (eds) Core Writing Team, Climate change 2014: Synthesis report. IPCC, Geneva, Switzerland.

[ref51] Johnson DM, McCulloh KA, Woodruff DR, Meinzer FC. (2012) Hydraulic safety margins and embolism reversal in stems and leaves: why are conifers and angiosperms so different? Plant Sci 195:48–53. 10.1016/j.plantsci.2012.06.010. 22920998

[ref52] Kinzinger L, Haberstroh S, Mach J, Weiler M, Orlowski N, Werner C. (2025) Continuous In-situ water stable isotopes reveal rapid changes in root water uptake by *Fagus sylvatica* during severe drought. Plant Cell Environ 48:7627–7639. 10.1111/pce.70055. 40637592 PMC12415413

[ref53] Knüver T, Bär A, Ganthaler A et al. (2022) Recovery after long-term summer drought: hydraulic measurements reveal legacy effects in trunks of *Picea abies* but not in *Fagus sylvatica*. Plant Biol 24:1240–1253. 10.1111/plb.13444. 35611757 PMC10084041

[ref54] Köcher P, Horna V, Leuschner C. (2013) Stem water storage in five coexisting temperate broad-leaved tree species: significance, temporal dynamics and dependence on tree functional traits. Tree Physiol 33:817–832. 10.1093/treephys/tpt055. 23999137

[ref55] Konings AG, Saatchi SS, Frankenberg C et al. (2021) Detecting forest response to droughts with global observations of vegetation water content. Glob Chang Biol 27:6005–6024. 10.1111/gcb.15872. 34478589 PMC9293345

[ref56] Leuschner C . (2020) Drought response of European beech (*Fagus sylvatica* L.)—a review. Perspect Plant Ecol Evol Syst 47:125576. 10.1016/j.ppees.2020.125576.

[ref57] Leuzinger S, Zotz G, Asshoff R, Körner C. (2005) Responses of deciduous forest trees to severe drought in Central Europe. Tree Physiol 25:641–650. 10.1093/treephys/25.6.641. 15805084

[ref58] Liu Z, Liu Q, Wei Z, Yu X, Jia G, Jiang J. (2021) Partitioning tree water usage into storage and transpiration in a mixed forest. For Ecosyst 8:72. 10.1186/s40663-021-00353-5.

[ref59] Liu Z, Ye L, Jiang J, Liu R, Xu Y, Jia G. (2023) Increased uptake of deep soil water promotes drought resistance in mixed forests. Plant Cell Environ 46:3218–3228. 10.1111/pce.14642. 37287350

[ref60] Mantova M, Herbette S, Cochard H, Torres-Ruiz JM. (2022) Hydraulic failure and tree mortality: from correlation to causation. Trends Plant Sci 27:335–345. 10.1016/j.tplants.2021.10.003. 34772610

[ref61] Martinetti S, Molnar P, Carminati A, Floriancic MG. (2025) Contrasting the soil–plant hydraulics of beech and spruce by linking root water uptake to transpiration dynamics. Tree Physiol 45. 10.1093/treephys/tpae158. PMC11761973 39658309

[ref63] Martín-Gómez P, Serrano L, Ferrio JP. (2017) Short-term dynamics of evaporative enrichment of xylem water in woody stems: implications for ecohydrology. Tree Physiol 37:511–522. 10.1093/treephys/tpw115. 27974650

[ref62] Martín-Gómez P, Rodríguez-Robles U, Ogée J et al. (2023) Contrasting stem water uptake and storage dynamics of water-saver and water-spender species during drought and recovery. Tree Physiol 43:1290–1306. 10.1093/treephys/tpad032. 36930058

[ref64] Matheny AM, Bohrer G, Garrity SR, Morin TH, Howard CJ, Vogel CS. (2015) Observations of stem water storage in trees of opposing hydraulic strategies. Ecosphere 6:1–13. 10.1890/ES15-00170.1.

[ref65] McDowell NG, Sapes G, Pivovaroff A et al. (2022) Mechanisms of woody-plant mortality under rising drought, CO2 and vapour pressure deficit. Nat Rev Earth Environ 3:294–308. 10.1038/s43017-022-00272-1.

[ref66] Milad M, Schaich H, Bürgi M, Konold W. (2011) Climate change and nature conservation in central European forests: a review of consequences, concepts and challenges. For Ecol Manag 261:829–843. 10.1016/j.foreco.2010.10.038.

[ref67] Montemagno A, Keim RF, López-Días V et al. (2023) Progressive enrichment in 18O and 2H in xylem water along sap flow paths in *Fagus sylvatica* trees. Ecohydrology 16:1–14. 10.1002/eco.2582.

[ref68] Nickel UT, Weikl F, Kerner R, Schäfer C, Kallenbach C, Munch JC, Pritsch K. (2018) Quantitative losses vs. qualitative stability of ectomycorrhizal community responses to 3 years of experimental summer drought in a beech–spruce forest. Glob Chang Biol 24:e560–e576. 10.1111/gcb.13957. 29063659

[ref69] Niinemets Ü . (2010) Responses of forest trees to single and multiple environmental stresses from seedlings to mature plants: past stress history, stress interactions, tolerance and acclimation. For Ecol Manag 260:1623–1639. 10.1016/j.foreco.2010.07.054.

[ref70] Nikolova PS, Bauerle TL, Häberle KH, Blaschke H, Brunner I, Matyssek R. (2020) Fine-root traits reveal contrasting ecological strategies in European beech and Norway spruce during extreme drought. Front Plant Sci 11:1–18. 10.3389/fpls.2020.01211. 32903505 PMC7438540

[ref71] North GB, Nobel PS. (1997) Drought-induced changes in soil contact and hydraulic conductivity for roots of *Opuntia ficus-indica* with and without rhizosheaths. Plant Soil 191:249–258. 10.1023/A:1004213728734.

[ref72] Novàk V, Simunek J, van Genuchten MT. (2000) Infiltration of water into soil with cracks. J Irrig Drain Eng 126:41–47. 10.1061/(ASCE)0733-9437(2000)126:1(41).

[ref73] Olesinski J, Lavigne MB, Krasowski MJ. (2011) Effects of soil moisture manipulations on fine root dynamics in a mature balsam fir (*Abies balsamea* L. Mill.) forest. Tree Physiol 31:339–348. 10.1093/treephys/tpr006. 21489968

[ref74] Paligi SS, Link RM, Hackmann CA, Coners H, Leuschner C. (2025) Water consumption of beech, spruce and Douglas fir in pure and mixed stands in a wet and a dry year – testing predictions of the iso/anisohydry concept. Sci Total Environ 970:178948. 10.1016/j.scitotenv.2025.178948. 40043649

[ref75] Phillips DL, Newsome SD, Gregg JW. (2005) Combining sources in stable isotope mixing models: alternative methods. Oecologia 144:520–527. 10.1007/s00442-004-1816-8. 15711995

[ref76] Poyatos R, Granda V, Flo V et al. (2021) Global transpiration data from sap flow measurements: the SAPFLUXNET database. Earth Syst Sci Data 13:2607–2649. 10.5194/essd-13-2607-2021.

[ref77] Prechsl UE, Burri S, Gilgen AK, Kahmen A, Buchmann N. (2015) No shift to a deeper water uptake depth in response to summer drought of two lowland and sub-alpine C3-grasslands in Switzerland. Oecologia 177:97–111. 10.1007/s00442-014-3092-6. 25273953

[ref78] Preisler Y, Hölttä T, Grünzweig JM, Oz I, Tatarinov F, Ruehr NK, Rotenberg E, Yakir D. (2022) The importance of tree internal water storage under drought conditions. Tree Physiol 42:771–783. 10.1093/treephys/tpab144. 34726242

[ref82] Pretzsch H, Schütze G, Uhl E. (2013) Resistance of European tree species to drought stress in mixed versus pure forests: evidence of stress release by inter-specific facilitation. Plant Biol 15:483–495. 10.1111/j.1438-8677.2012.00670.x. 23062025

[ref81] Pretzsch H, Rötzer T, Matyssek R, Grams TEE, Häberle KH, Pritsch K, Kerner R, Munch JC. (2014) Mixed Norway spruce (Picea abies [L.] Karst) and European beech (*Fagus sylvatica* [L.]) stands under drought: from reaction pattern to mechanism. Trees Struct Funct 28:1305–1321. 10.1007/s00468-014-1035-9.

[ref80] Pretzsch H, Grams T, Häberle KH, Pritsch K, Bauerle T, Rötzer T. (2020) Growth and mortality of Norway spruce and European beech in monospecific and mixed-species stands under natural episodic and experimentally extended drought. results of the KROOF throughfall exclusion experiment. Trees Struct Funct 34:957–970. 10.1007/s00468-020-01973-0.

[ref83] R Development Core Team (2008) R: a language and environment for statistical computing. R Foundation for Statistical Computing, Vienna, Austria. http://www.R-project.org.

[ref84] RStudio Team (2015) RStudio: Integrated Development for R. http://www.rstudio.com/

[ref85] Ruehr NK, Grote R, Mayr S, Arneth A. (2019) Beyond the extreme: recovery of carbon and water relations in woody plants following heat and drought stress. Tree Physiol 39:1285–1299. 10.1093/treephys/tpz032. 30924906 PMC6703153

[ref86] Schuldt B, Buras A, Arend M et al. (2020) A first assessment of the impact of the extreme 2018 summer drought on central European forests. Basic Appl Ecol 45:86–103. 10.1016/j.baae.2020.04.003.

[ref87] Seeger S, Weiler M. (2021) Temporal dynamics of tree xylem water isotopes: in situ monitoring and modeling. Biogeosciences 18:4603–4627. 10.5194/bg-18-4603-2021.

[ref88] Spiecker H . (2000) Growth of Norway spruce (*Picea abies* [L.] Karst.) under changing environmental conditions in Europe. In: Emil Klimo, Herbert Hager and Jiøí Kulhavý (eds), Spruce monocultures in Central Europe-problems and prospects. Joensuu, European Forest Institute, pp 11–26.

[ref89] Tomasella M, Beikircher B, Häberle K-H, Hesse B, Kallenbach C, Matyssek R, Mayr S. (2018) Acclimation of branch and leaf hydraulics in adult *Fagus sylvatica* and *Picea abies* in a forest through-fall exclusion experiment. Tree Physiol 38:198–211. 10.1093/treephys/tpx140. 29177459

[ref90] Tomasella M, Häberle K-H, Nardini A, Hesse B, Machlet A, Matyssek R. (2017) Post-drought hydraulic recovery is accompanied by non-structural carbohydrate depletion in the stem wood of Norway spruce saplings. Sci Rep 7:14308. 10.1038/s41598-017-14645-w. 29085007 PMC5662761

[ref91] Trugman AT, Detto M, Bartlett MK, Medvigy D, Anderegg WRL, Schwalm C, Schaffer B, Pacala SW. (2018) Tree carbon allocation explains forest drought-kill and recovery patterns. Ecol Lett 21:1552–1560. 10.1111/ele.13136. 30125446

[ref92] Tschaplinski TJ, Blake TJ. (1985) Effects of root restriction on growth correlations, water relations and senescence of alder seedlings. Physiol Plant 64:167–176. 10.1111/j.1399-3054.1985.tb02331.x.

[ref94] Volkmann THM, Weiler M. (2014) Continual in situ monitoring of pore water stable isotopes in the subsurface. Hydrol Earth Syst Sci 18:1819–1833. 10.5194/hess-18-1819-2014.

[ref93] Volkmann THM, Kühnhammer K, Herbstritt B, Gessler A, Weiler M. (2016) A method for in situ monitoring of the isotope composition of tree xylem water using laser spectroscopy. Plant Cell Environ 39:2055–2063. 10.1111/pce.12725. 27260852

[ref95] Wagner Y, Volkov M, Nadal-Sala D, Ruehr NK, Hochberg U, Klein T. (2023) Relationships between xylem embolism and tree functioning during drought, recovery, and recurring drought in Aleppo pine. Physiol Plant 175:e13995. 10.1111/ppl.13995. 37882273

[ref96] Waring RH, Whitehead D, Jarvis PG. (1979) The contribution of stored water to transpiration in scots pine. Plant Cell Environ 2:309–317. 10.1111/j.1365-3040.1979.tb00085.x.

[ref97] Werner C, Schnyder H, Cuntz M et al. (2012) Progress and challenges in using stable isotopes to trace plant carbon and water relations across scales. Biogeosciences 9:3083–3111. 10.5194/bg-9-3083-2012.

[ref98] West AG, Patrickson SJ, Ehleringer JR. (2006) Water extraction times for plant and soil materials used in stable isotope analysis. Rapid Commun Mass Spectrom 20:1317–1321. 10.1002/rcm.2456. 16555369

[ref99] Zang U, Goisser M, Grams TEE, Haberle KH, Matyssek R, Matzner E, Borken W. (2014) Fate of recently fixed carbon in European beech (*Fagus sylvatica*) saplings during drought and subsequent recovery. Tree Physiol 34:29–38. 10.1093/treephys/tpt110. 24420388

[ref101] Zarebanadkouki M, Carminati A. (2014) Reduced root water uptake after drying and rewetting. J Plant Nutr Soil Sci 177:227–236. 10.1002/jpln.201300249.

[ref100] Zarebanadkouki M, Ahmed MA, Carminati A. (2016) Hydraulic conductivity of the root-soil interface of lupin in sandy soil after drying and rewetting. Plant Soil 398:267–280. 10.1007/s11104-015-2668-1.

[ref102] Zheng C, Bochmann H, Liu Z, Kant J, Schrey SD, Wojciechowski T, Postma JA. (2023) Plant root plasticity during drought and recovery: what do we know and where to go? Front Plant Sci 14:1–14. 10.3389/fpls.2023.1084355. PMC10061088 37008469

[ref103] Zwetsloot MJ, Bauerle TL. (2021) Repetitive seasonal drought causes substantial species-specific shifts in fine-root longevity and spatio-temporal production patterns in mature temperate forest trees. New Phytol 231:974–986. 10.1111/nph.17432. 33908081

[ref104] Zwetsloot MJ, Goebel M, Paya A, Grams TEE, Bauerle TL. (2019) Specific spatio-temporal dynamics of absorptive fine roots in response to neighbor species identity in a mixed beech–spruce forest. Tree Physiol 39:1867–1879. 10.1093/treephys/tpz086. 31504991

