# Supplementary material for: Delayed uptake and intra-tree distribution of 2H-labeled irrigation water after repeated experimental summer drought in mature spruce compared with beech
Source: Tree Physiol. 2025 Dec 10;46(1):tpaf153. doi: 10.1093/treephys/tpaf153 (PMC12797068; doi:10.1093/treephys/tpaf153)
Supplement: tpaf153_Hesse_KROOF_deuterium_labeling_supplement [file tpaf153_hesse_kroof_deuterium_labeling_supplement.docx]

**Title**

Delayed uptake and intra-tree distribution of ^2^H-labelled irrigation water after repeated experimental summer drought in mature spruce compared to beech

**Running head**

Water distribution upon irrigation after repeated drought in mature beech and spruce.

**Authors names:**

Benjamin D. Hesse^1,2^, Benjamin D. Hafner^3^, Timo Gebhardt^4^, Stefan Seeger^5,6^, Kyohsuke Hikino^2,7^, Eva Stempfle^2^, Regina Seiler^2^, Karl-Heinz Häberle^8^, Markus Weiler^6^, Thorsten E. E. Grams^2^

**Authors’ affiliation**

1. University of Natural Resources and Life Sciences, Department of Ecosystem Management, Climate and Biodiversity, Institute of Botany, Gregor-Mendel-Straße 33, 1180 Vienna, Austria.
2. Technical University of Munich, School of Life Sciences - Chair for Land Surface-Atmosphere Interactions - AG Ecophysiology of Plants, Hans-Carl-von-Carlowitz Platz 2, 85354 Freising, Germany.
3. Technical University of Munich, School of Life Sciences, Soil Biophysics & Environmental Systems, Hans-Carl-von-Carlowitz Platz 2, 85354 Freising, Germany.
4. Technical University of Munich, School of Life Sciences, Forest and Agroforest Systems, Hans-Carl-von-Carlowitz Platz 2, 85354 Freising, Germany.
5. University of Göttingen, Department of Crop Sciences, Soil Physics, Grisebachstraße 6 37077 Göttingen
6. Chair of Hydrology, Faculty of Environment and Natural Resources, University of Freiburg, Fahnenbergplatz, 79098 Freiburg, Germany.
7. Swedish University of Agricultural Sciences, Department of Forest Ecology and Management, Skogsmarksgränd 17, 907 36 Umeå, Sweden.
8. Technical University of Munich, School of Life Sciences, Chair of Restoration Ecology, Emil-Ramann-Str. 6, 85354 Freising, Germany.

# Supplemental material

Table S 1: Daily average temperature (in °C), vapor pressure deficit (VPD, in kPa) and global solar radiation (in W m^-2^) of the first 15 days after labeling of all 3 campaigns

| Daily mean | Temperature [°C] | VPD [kPa] | Global radiation [W m^-2^] |
| --- | --- | --- | --- |
| Campaign 1 | 21.9 ± 3.8 | 1.2 ± 0.4 | 465 ± 109 |
| Campaign 2 | 17.0 ± 2.4 | 0.7 ± 0.4 | 400 ± 110 |
| Campaign 3 | 18.5 ± 3.4 | 0.8 ± 0.5 | 401 ± 96 |

Table S 2: Sampling table with timepoints for δ^2^H measurement in different tissues, with D0 = day of irrigation/labeling. Soil = soil cores with samples from 0-70 cm in 10 cm segments, Stem = xylem sapwood at DBH with in-situ probes, Twig = twig xylem from the sun canopy and Leaf = leaves from sum canopy.

| **Tissue** | **D-6** | **D-1** | **D0** | **D1** | **D2** | **D4** | **D7** | **D15** |
| --- | --- | --- | --- | --- | --- | --- | --- | --- |
| **Soil** | x | x | x | x | x | x | x | x |
| **Stem** |  |  | x | x | x | x | x | x |
| **Twig** | x |  |  |  |  |  |  | x |
| **Leaf** | x | x | x | x | x | x | x | x |


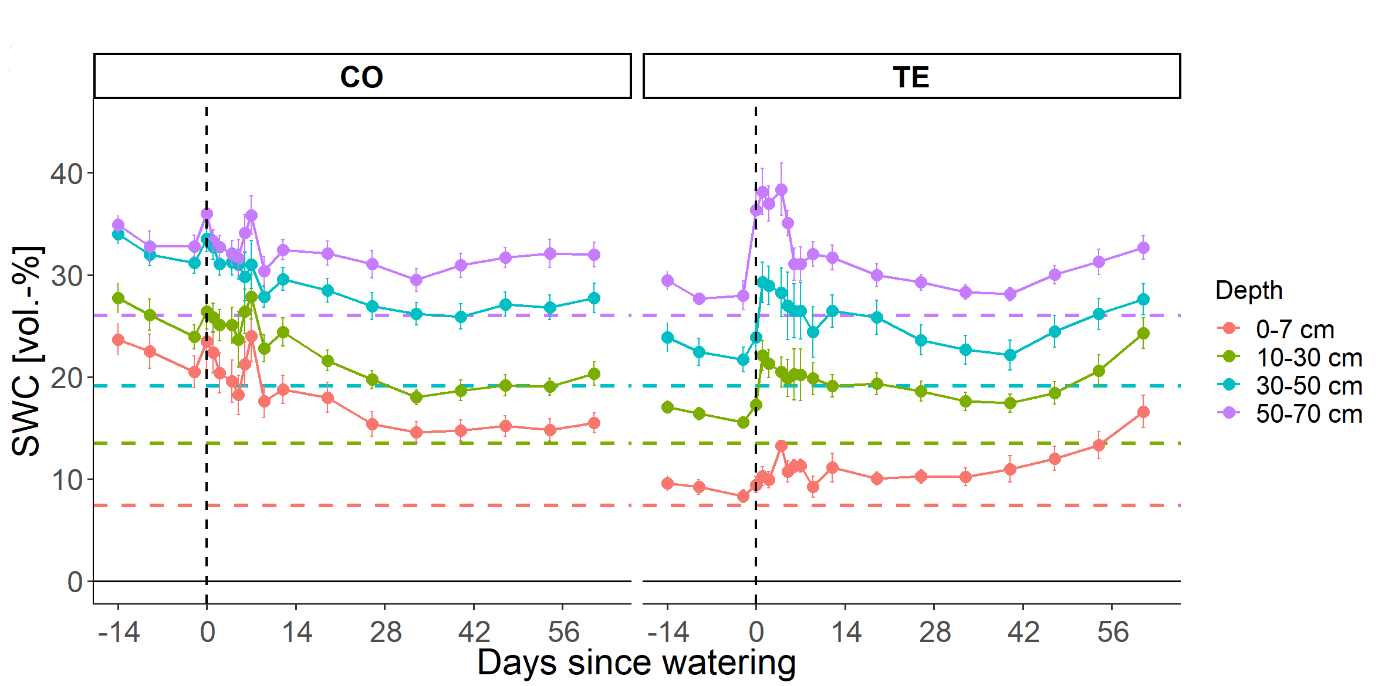


Figure S 1:Soil water content (SWC in vol.-%) in control (CO) and throughfall-exclusion (TE) of different soil depths (0-7 cm = orange, 10-30 cm = green, 30-50 cm = blue and 50 to 70 cm = purple, horizontal dashed lines = PWP of different depths according to Grams et al. (2021) and vertical dashed line = day of irrigation))


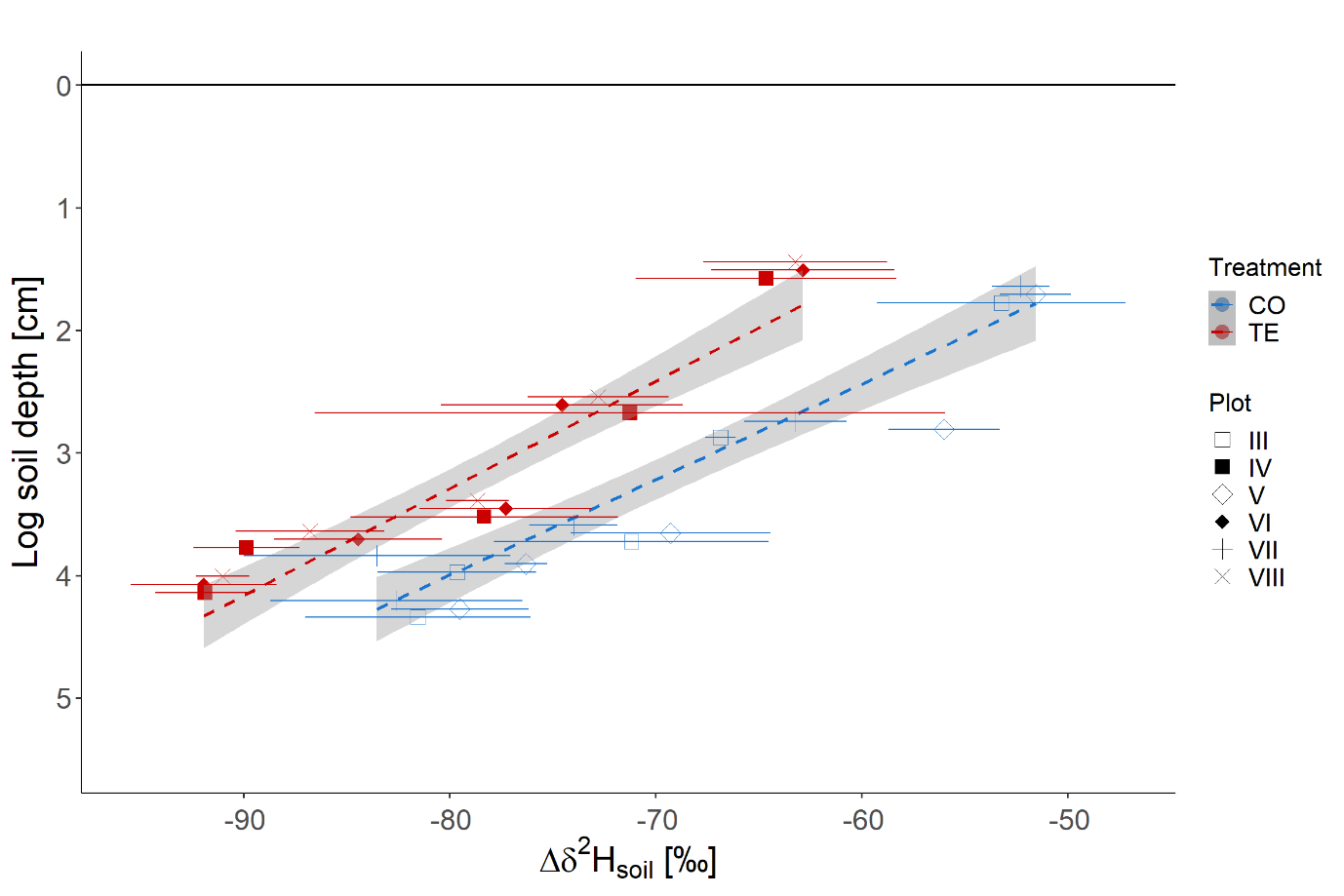


Figure S 2: Linear regression between δ^2^H_soil_ and the logarithmic soil depth for control (CO, blue, R^2^=0.91) and throughfall-exclusion (TE, red, R^2^=0.92) before the start of the irrigation to assess the mean water uptake depth. Different symbols reflect the plots used in this analysis. Symbols show the mean ± 1SE.
